# Does the Lectin Complement Pathway Link Kawasaki Disease and SARS-CoV-2?

**DOI:** 10.3389/fimmu.2020.604512

**Published:** 2021-01-14

**Authors:** Anastasia Polycarpou, Sofia Grigoriadou, Linda Klavinskis, Steven Sacks

**Affiliations:** ^1^ School of Immunology and Microbial Sciences, King’s College London, London, United Kingdom; ^2^ Immunology Department, Royal London Hospital, Barts Health NHS Trust, London, United Kingdom

**Keywords:** complement, lectin pathway, SARS-CoV-2, multisystem inflammatory syndrome, Kawasaki disease, children, COVID-19, paediatric

## Introduction

Out of the mire of the COVID-19 pandemic, caused by the novel coronavirus SARS-CoV-2, a new clue on pathogenesis has emerged regarding multisystem inflammatory disease in children with evidence of endotheliitis and microvascular thrombosis, with striking similarities to Kawasaki disease. Here we consider whether the complement system could underpin these connections, in particular the lectin pathway - the most recent and perhaps least understood of the three main activation pathways known to unleash the complement cascade. We have evaluated the published evidence linking the complement system with both Kawasaki disease and COVID-19 associated acute respiratory distress syndrome (ARDS) and we conclude that the complement system could be a therapeutic target for the Kawasaki-like syndrome triggered by SARS-CoV-2.

The complement cascade has a significant role in host defence. Activation of complement *via* the classical and alternative pathways has long been recognised. More recently, the lectin complement pathway has defined another means of pathogen recognition, *via* carbohydrate motifs on the pathogen surface that can bind vertebrate lectins, resulting in enzymatic activation of complement and also the coagulation system on endothelial surfaces to cause thrombosis (1). Pertinently, the surface structures of SARS-CoV-2 are rich in carbohydrate residues recognised by lectins (2).

## Lectin Pathway Involvement in COVID-19

**Figure 1:**
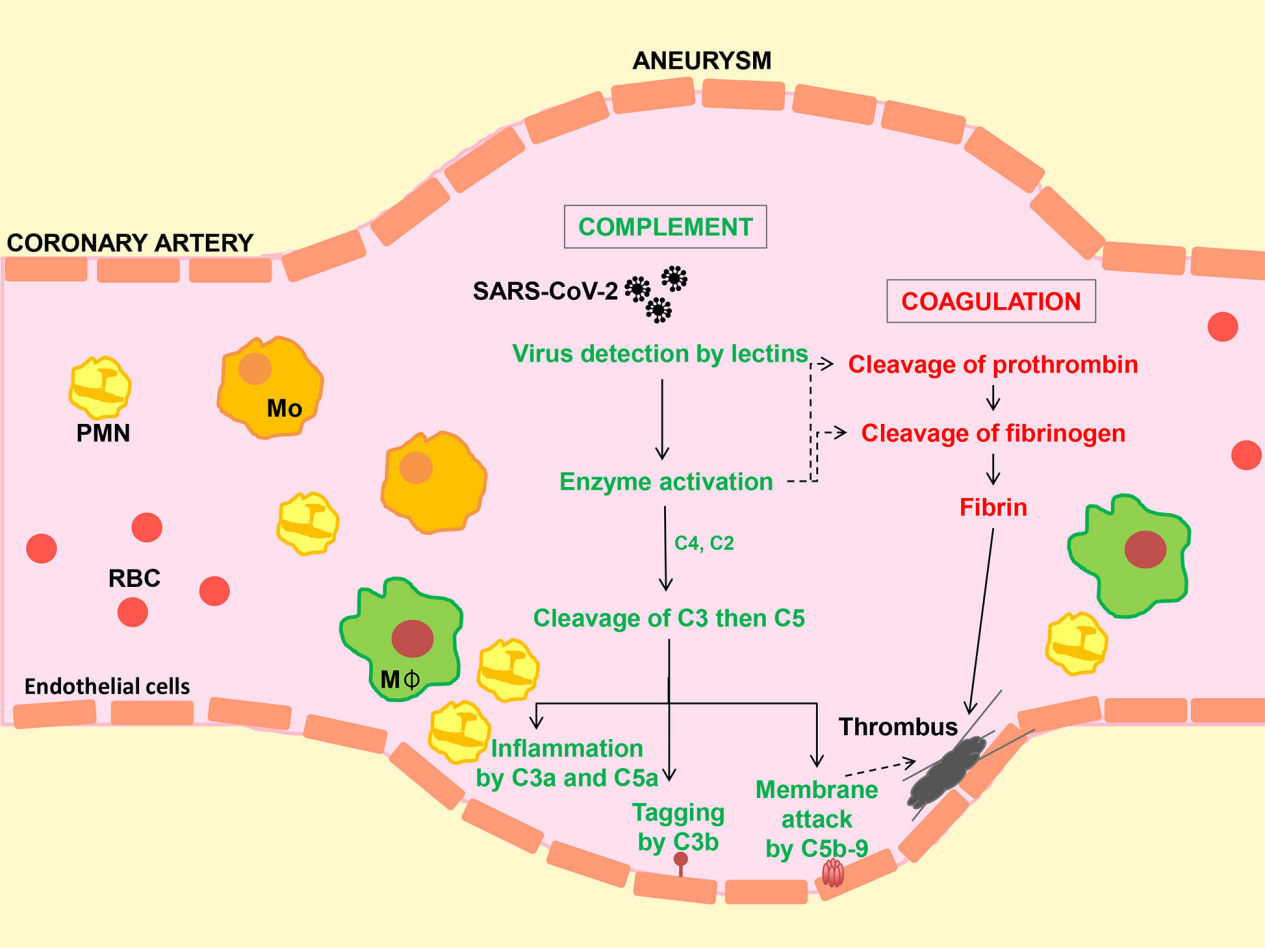
Hypothetical model to illustrate activation of the complement system, inflammation and coagulation following SARS-CoV-2 infection. Virus enters the circulation from the lung alveolar space. Carbohydrate structures in the viral envelope are detected by circulating lectins (e.g. collectin-11 and mannan-binding lectin, MBL). Enzymes bound to collectins (known as MBL-associated serine proteases or MASPs) lead to the cleavage of complement C3 (directly or *via* C4 and C2) followed by C5 to generate biologically activate fragments (C3a, C3b, C5a and C5b) and membrane attack complex (C5b-9). Membrane damage caused by the actions of C5a and C5b-9 and inflammatory cell infiltration on the blood vessel wall promotes clot (thrombus) formation by the coagulation pathway, while in addition, lectin-MASP complexes can directly activate the coagulation cascade. Reports of microscopic tissue examination in cases of COVID-19 show evidence of small and medium size vessel injury (vasculitis) and thrombus formation in the presence of SARS-CoV-2, MBL, MASP, C3b and C5b-9, suggesting a causal relationship as depicted. Mo, Monocyte; MΦ, macrophage; PMN, polymorphonuclear neutrophil; RBC, Erythrocyte.

The hallmarks of lectin pathway involvement—increased detection of Mannose binding lectin (MBL) and MBL-associated serine protease-2 (MASP-2) alongside cleavage products of complement C4 and C3—are found co-localised with SARS-CoV-2 in post-mortem lung tissue from adult COVID-19 patients (3). The alveolar epithelium, a primary target of SARS-CoV-2, locally produces complement-activating lectins such as collectin-11 and ficolin-1 providing a tentative site for complement deposition. Indeed, lectin pathway components (MBL, MASP-2, C4d, and C3d) are also co-localised with the SARS-CoV-2 envelope protein in adjacent blood vessels, where endotheliitis and thrombus formation are evident (see **Figure 1**) (3). It should be emphasised that activation of the lectin pathway through MBL-MASP and ficolin-MASP binding to glycan structures, which are present on the SARS-CoV-2 envelope, may lead to the generation of blood clots similar to those generated by thrombin (4). Moreover, elevated plasma collectin-11 levels have been associated with the risk of disseminated intravascular coagulation (DIC) (5). These findings provide the first circumstantial evidence of complement involvement in adult COVID-19. Although not proving causation, they have provided rationale for targeting the complement system in COVID-19 (6) and the initiation of clinical trials of complement inhibitors.

## Comparison of Multisystem Inflammatory Syndrome in Children (MIS-C) Following SARS-CoV-2 Infection With Kawasaki Disease

While childhood COVID-19 is usually asymptomatic or mild, several recent published reports document a hyper-inflammatory syndrome associated with thrombotic tendency, multi-organ involvement and a high rate of positivity for SARS-CoV-2 (7–13). This syndrome has resemblance to Kawasaki disease, first recognised in Japan in 1967, which is associated with several viral infections including coronaviruses (14) and presents as an acute vasculitis of children under five, characterised by fever, lymphadenopathy and coronary artery involvement in up to 25% (15). In comparison, patients with the Kawasaki-like paediatric variant of COVID-19 are generally older, often presenting with severe respiratory and gastrointestinal symptoms. Two recent studies that reported a large number of patients with confirmed or suspected cases of multisystem inflammatory syndrome noted positivity for SARS-CoV-2 by RT-PCR or antibody testing in 70% and 99% respectively (11, 12). The majority of children in all studies reported to date had increased markers of myocardial injury, many had echocardiogram abnormalities and/or prominent echogenic coronary arteries while 14% in the English study (8) and 9% in the two large American cohorts (11, 12) also had coronary aneurysms. In addition, increased coagulation markers were noted in a significant proportion of patients when measured, while acute kidney injury occurred in 22% of admitted children in the English cohort (9). Thus, the clinical and laboratory findings in multisystem inflammatory syndrome in children (MIS-C) following infection with SARS-CoV-2 have considerable overlap with Kawasaki disease and with adult COVID-19.

## Links Between MIS-C and Kawasaki Disease With the Complement System

A viral etiology is implicated in adult and paediatric COVID-19 and Kawasaki disease. The wide range of viruses including SARS-Coronaviruses (CoV) interacting with carbohydrate-recognition molecules such as collectin-11 (16) and MBL (17) makes it highly likely that the lectin complement pathway is triggered. Moreover, MBL gene polymorphism associates with Kawasaki disease and its cardiovascular abnormalities (18–20) and MBL has been directly implicated in the pathogenesis of a murine model of vasculitis imitating Kawasaki disease (21). A dual role for MBL has been proposed in Kawasaki disease depending on the age of the patient, with a pathogenic role favoring disease progression and endothelial damage in older children (20). Additionally, plasma MASP-1 concentration at the onset of the illness was predictive of the length of recovery time for coronary artery lesions (22). There is evidence that activation of MASP-1 in the early stage of Kawasaki disease provokes a local vascular inflammatory reaction, resulting in the consumption of MASP-1 (22). Another lectin pathway component, ficolin-1, demonstrates reduced serum levels after successful intravenous immunoglobulin (IVIG) treatment in Kawasaki patients (23). This study provided support for direct interaction of IVIG with ficolin-1, giving rise to the captivating idea that at least one mechanism of treatment effectiveness of IVIG in Kawasaki disease is due to reduction of ficolin-1 level in the serum (23).

Other pathways of complement activation may be involved. Complement activation by immune complexes in Kawasaki disease suggests dependence on the classical pathway of complement activation in the development of vasculitis (24). However, elevation of plasma levels of activation marker C4d in patients with Kawasaki syndrome, which has been previously reported to be diagnostic of the classical pathway (24), could also be downstream of the lectin pathway since C4 is a component of both complement pathways (25). C-reactive protein (CRP) may further enhance the localised deposition of complement in the vessel wall, since gross elevation of CRP occurs in the acute phase of Kawasaki disease (and COVID-19) and this protein can mediate classical pathway activation of complement (26, 27). A study in Kawasaki disease demonstrated that complement factor B (essential for alternative pathway activation) is likely to have a contributory effect (28). Other proteomic studies have identified strong associations of the complement and coagulation cascades with Kawasaki disease (29). Multiple different components of innate immunity may be active during MIS-C, Kawasaki syndrome and COVID-19 such as type I interferon (IFN) (30, 31), which may be a downstream effect of complement activation or may occur independently. The lectin pathway could therefore be the initial immune trigger after engaging the virus; initiating the complement cascade, while induction of type I IFN could be a secondary response. Independent of which of the two systems is activated first, thrombotic microangiopathy may be a resulting manifestation (32).

## Discussion

At this early stage in our understanding of how SARS-CoV-2 leads to hyperinflammatory syndrome, the evidence linking complement activation with endothelial injury and thrombosis is undeniable though largely circumstantial. More-definitive experimental data is needed to support complement-based therapeutic approaches and to inform on the most appropriate therapeutic targets and whether these involve ligand-recognition (lectin and classical) or amplification (alternative) pathways, or core components (C3 and C5) or the terminal effector pathway (i.e. C5a or C5b-9). It is hoped that this opinion article will stimulate early interest in these possibilities. The pandemic of SARS-CoV-2 disease and its aggressive presentation in a subgroup of children also brings into line a better understanding of the pathogenesis of sporadic Kawasaki disease and the potential for targeting the complement system in both conditions.

## Author Contributions

All authors participated in reviewing the literature. AP, SS and LK contributed in writing and editing the manuscript. SG provided information on paediatric clinical cases and contributed to editing. All authors contributed to the article and approved the submitted version.

## Funding

Previously published research by our laboratories was supported by the UK Medical Research Council (grants MR/J006742/1, MR/L020254/1, G1001141, MR/J004553/1, MR/M007871/1 and MR/L012758/1), the National Institute for Health Research (NIHR) Biomedical Research Centre based at Guy’s and St Thomas’s NHS (grant RE12572), The Bill and Melinda Gates Foundation, Seattle, WA grant number 38639 and European Union Marie Curie Initial Training Network (UniVacFlu) grant number 607690 to LK.

## Conflict of Interest

SS consults for UCB, Omeros and Alexion Pharmaceuticals Inc. on therapeutic targets in the complement system.

The remaining authors declare that the research was conducted in the absence of any commercial or financial relationships that could be construed as a potential conflict of interest.
